# Microstructural integrity of white matter tracts amongst older fallers: A DTI study

**DOI:** 10.1371/journal.pone.0179895

**Published:** 2017-06-28

**Authors:** Yoke Queen Wong, Li Kuo Tan, Pohchoo Seow, Maw Pin Tan, Khairul Azmi Abd Kadir, Anushya Vijayananthan, Norlisah Ramli

**Affiliations:** 1 University Malaya Research Imaging Centre, Department of Biomedical Imaging, Faculty of Medicine, University of Malaya, Kuala Lumpur, Malaysia; 2 Department of Diagnostic Imaging, Hospital Selayang, Batu Caves, Selangor, Malaysia; 3 Department of Geriatric Medicine, University Malaya Medical Centre, Kuala Lumpur, Malaysia; Istituto Di Ricerche Farmacologiche Mario Negri, ITALY

## Abstract

**Objectives:**

This study assesses the whole brain microstructural integrity of white matter tracts (WMT) among older individuals with a history of falls compared to non-fallers.

**Methods:**

85 participants (43 fallers, 42 non-fallers) were evaluated with conventional MRI and diffusion tensor imaging (DTI) sequences of the brain. DTI metrics were obtained from selected WMT using tract-based spatial statistics (TBSS) method. This was followed by binary logistic regression to investigate the clinical variables that could act as confounding elements on the outcomes. The TBSS analysis was then repeated, but this time including all significant predictor variables from the regression analysis as TBSS covariates.

**Results:**

The mean diffusivity (MD) and axial diffusivity (AD) and to a lesser extent radial diffusivity (RD) values of the projection fibers and commissural bundles were significantly different in fallers (p < 0.05) compared to non-fallers. However, the final logistic regression model obtained showed that only functional reach, white matter lesion volume, hypertension and orthostatic hypotension demonstrated statistical significant differences between fallers and non-fallers. No significant differences were found in the DTI metrics when taking into account age and the four variables as covariates in the repeated analysis.

**Conclusion:**

This DTI study of 85 subjects, do not support DTI metrics as a singular factor that contributes independently to the fall outcomes. Other clinical and imaging factors have to be taken into account.

## Introduction

Falls are common in the older population. The annual incidence of falls is nearly 30% in individuals 65 years and above and the number is expected to increase further as the population ages [1]. Falls in older people lead to physical disability, psychosocial problems, deterioration in quality of life and reduced survival [2, 3]. It often results in hospitalization and institutionalization with expensive medical costs [4]. Falls in the elderly are associated with numerous risk factors, e.g. physical limitation, gait and balance deficits, visual impairment, chronic medical illness, neurological disorders and postural hypotension [5, 6].

In the LADIS study, Baezner et al (2008) concluded that a strong association exists between the severity of age-related white matter (WM) changes with gait and motor compromise [7]. Blahak et al (2009) have reported that the severity of WM changes were significantly associated with balance disturbances and falls, with periventricular and deep frontal WM changes found to be independent predictors for both balance disturbances and falls [8]. Nevertheless, a recent diffusion tensor imaging (DTI) study reveals that in older subjects with small vessel disease, disruption of WM integrity in both (a) WML that appears as hyperintensities on T2-weighted MRI images as well as (b) “normal-appearing WM”, are associated with gait disturbances [9]. Therefore DTI enables us to detect loss of integrity even in the “normal-appearing WM” on conventional MRI images.

An additional advantage of DTI is the ability to study the integrity of individual white matter tracts (WMT) [10], therefore allowing for better localization of WM pathologies. The integrity of the WMT can be quantified using fractional anisotropy (FA) and mean/ axial/ radial diffusivity (MD/ AD/ RD) as the loss of integrity will cause the loss of anisotropy and an increased diffusivity of water molecules. A DTI study by Bhadelia et al (2009) demonstrates that loss of WM integrity in the genu of corpus callosum is an important marker of gait problems in older individuals [11]. In a recent DTI study based on tract-based spatial statistics (TBSS) analysis, older individuals with gait and balance impairments determined with an established scale have WM abnormalities in specific locations in the brain believed to be responsible for maintenance of normal gait [12].

Studies determining the integrity of the WMT among older fallers remain limited, with many studies evaluating surrogate balance and gait outcomes rather than comparing WMT changes with actual fall outcomes. To our knowledge, there has been no similar whole-brain analysis study in an Asian population. We, therefore, conducted a case-control comparison of WMT changes between older fallers with recurrent or injurious falls and non-fallers in an urban Malaysian population consisting of various racial backgrounds. We also took into account the various confounding factors contributing to falls to assess the importance of DTI as an independent diagnostic measure.

## Material and methods

### Recruitment of participants

A total of 85 subjects were recruited for the study over a period of 2 years which included 43 patients with falls and 42 healthy non-fallers. Fallers were recruited from among participants of a separate randomised controlled trial—the Malaysian Falls Assessment and Intervention Trial (MyFAIT) [13]. These participants were urban-dwelling older adult fallers recruited from the primary care unit, geriatric clinic, the Accident & Emergency department, and through referrals from other specialties. Participants were included if they satisfied two criteria: (i) being 65 years or older in age with (ii) a history of two or more falls, or one injurious fall in the past 12 months. Participants were excluded if they had any of the following: (i) clinically diagnosed dementia based on the ICD-10 criteria (International Classification of Diseases, 10th Edition (ICD-10) definition) as determined by a consultant geriatrician with at least 10 years of experience, (ii) severe physical disabilities (iii) major psychiatric illnesses, psychosis or brain damage, or (iv) contraindication to MRI. Non-fallers were those with no history of falls in at least 12 months, and were recruited from senior citizen groups from within the hospital catchment population. Verbal and written informed consent was obtained from all patients before recruitment. The study was approved by the Medical Ethics Committee of University Malaya Medical Center in 2012 (Ethics approval no. 943.21).

### Clinical assessment

All participants received a baseline clinical assessment conducted by trained researchers and a consultant geriatrician. Gait and balance performance were assessed using Functional Reach (FR) and Timed Up-and-Go (TUG) tests [13]. FR was used to identify change in balance performance over time while TUG was a composite measure of walking speed, limb girdle strength and dynamic balance. Other clinical variables assessed included age, gender, ethnicity, and history of diabetes, hypertension, stroke, orthostatic hypotension, syncopal events, carotid sinus syndrome, visual impairment, osteoarthritis and peripheral neuropathy. Blood pressure was measured using an digital sphygmomanometer first in the supine position after at least 10 minutes’ rest, and at the first, second and third minutes after standing. Orthostatic hypotension was diagnosed if a systolic or diastolic blood pressure reduction of at least 20mmHg or 10mmHg respectively or both were present [14].

### Magnetic resonance imaging acquisitions

All MR examinations were performed on a clinical 3.0 Tesla Signa® HDx MR System (GE Healthcare, Milwaukee, Wisconsin, USA) equipped with a dedicated 8-channel high definition head coil. The imaging protocols included: (i) Axial T1-weighted 3D fast spoiled gradient echo (3D FSPGR) with imaging parameters of TR = minimum 6.7ms, TE = minimum 1.9ms, range 1.9–11.0ms, FOV = 31mm, matrix = 256 x 256, isovoxel, thickness = 1.2mm and image scan time of 3mins 47s; (ii) Coronal T2-weighted fluid-attenuated inversion recovery sequence (FLAIR) with TR = 1600ms, TE = minimum 9.9ms, range 9.8–68.5mms, TI = 920ms, FOV = 24mm, matrix = 512 x 320, thickness = 5.0mm, spacing = 2mm and image scan time of 2mins 57s; and (iii) DTI datasets using single-shot spin-echo echo-planar imaging (EPI) with TR = 13000ms, TE = 81.2ms, FOV = 24mm, matrix = 128 x 128, thickness = 3.0 mm, gradient encoding along 32 non-collinear directions and diffusion-weighted factor, *b* = 700 s/mm^2^ and image scan time of 7mins 22s.

### Diffusion tensor imaging (DTI) analysis

Voxel-wise, whole brain tract-based spatial statistics (TBSS) were carried out to identify tracts of interest that showed significant differences between fallers and non-fallers (FSL v5.0.6 University of Oxford, UK) [15]. Standard preprocessing was applied to all volumes, including eddy current correction, brain masking, and linear fitting of the diffusion tensors. TBSS-specific steps [16] included non-linear registration of all images to the FMRIB58_FA standard-space image, and FA skeletonization with an FA threshold of 0.2. Randomise, a subtool in TBSS was used to carry out voxel-wise statistical analysis. The analysis was carried out with 10000 permutations using threshold-free cluster enhancement with 2D optimization as correction for multiple comparison across voxels.

### Voxel-based morphometry (VBM) analysis

**Fig 1:**
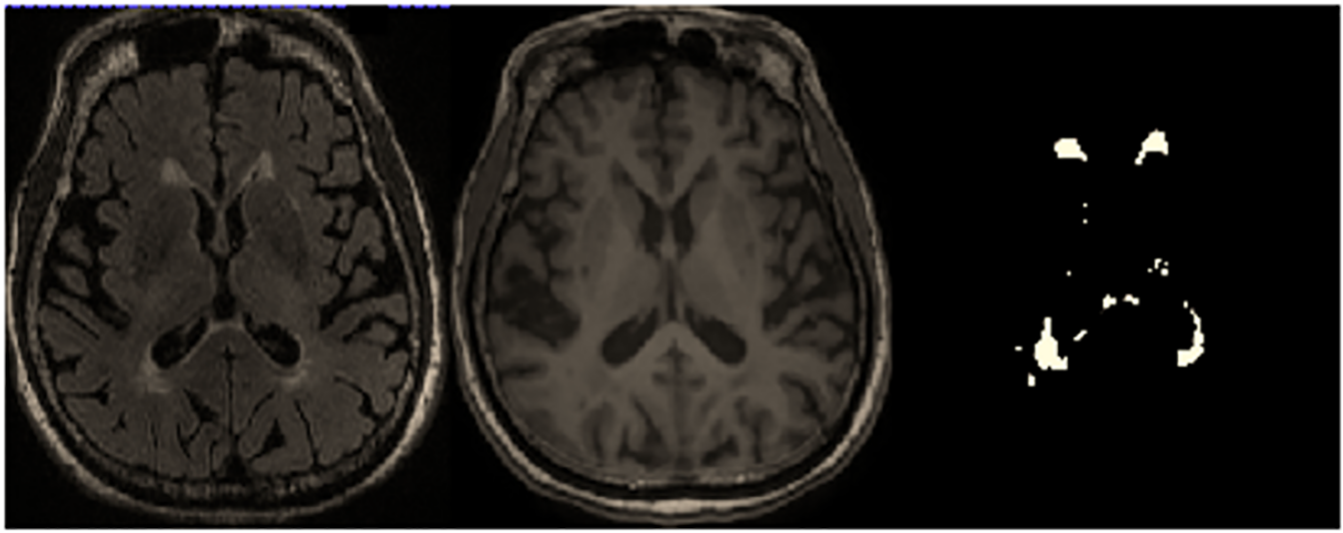
The VBM analysis. Axial T2-Weighted FLAIR Image, T1-Weighted FSPGR Image and lesions segmented using LST of SPM8.

Axial T1-weighted 3D FSPGR images were used for whole brain volume measurement. Voxel-based morphometry (VBM) analysis was computed using Christian Gaser's VBM8 toolbox [17] with default parameters, running within the Statistical Parametric Mapping software package version 8 (SPM8) [18]. The images were first bias-corrected for MRI field inhomogeneity, then registered to the brain averages of the subjects using linear and non-linear transformations, and tissue-classified into grey matter (GM), white matter (WM) and cerebrospinal fluid (CSF) within the same generative model. Total brain volume is the sum of the generated WM and GM volumes. After the pre-processing steps using SPM8 and its extension VBM8 as described above, the WML segmentation and total lesion volume (TLV) calculation were subsequently performed using the Lesion Segmentation Toolbox (LST) for SPM8 (Fig 1) [19]. The algorithm of LST operates in the space of the original T1-weighted image, known as the native space. The bias-corrected FLAIR image was co-registered to the space of the native T1 image. Each voxel in the native T1 image was assigned to 1 of 3 classes: GM, WM or CSF. The FLAIR intensity distributions were calculated for the respective 3 classes to detect hyperintense FLAIR outliers, which are further weighted according to their spatial probability of being WM lesion [20, 21].

### Statistical analysis

Individual statistical analysis for demographic, clinical and imaging characteristics was performed using computerized statistic software SPSS version 22.0 (Illinois, USA). Mann-Whitney and Chi-square tests were used for numerical and categorical variables respectively. A p-value of < 0.05 was considered to be statistically significant.

### Multiple logistic regression analysis

Multiple binary logistic regression analysis was carried out using fallers or non-fallers as the dependent variable, and demographic, clinical, and imaging characteristics as independent variables. Known risk factors for falls which showed significant differences in the univariate analysis were selected for the regression analysis. These factors included age, TUG, FR, WMLV, hypertension, orthostatic hypotension, and syncopal events (Table 1). The regression analysis was carried out using the stepwise backward likelihood ratio (backward LR) selection method. The TBSS analysis was then repeated, but this time including all significant predictor variables from the regression analysis as TBSS covariates. This was to investigate whether the DTI metrics contributed significantly to the outcomes independent of the covariates.

**Table 1:** Demographic, clinical and imaging characteristics of fallers and non-fallers at baseline.

		fallers (n = 43)				non-fallers (n = 42)				
Characteristics		Median	Mean	SD	Range	Median	Mean	SD	Range	p-values
Age (years)		74.0	73.21	4.26	65–83	71.0	71.52	4.55	65–84	0.027*
TUG (seconds)		13.38	17.42	10.55	7–62	11.0	11.97	3.55	6.7–22	0.003*
FR (cm)		21.0	19.97	7.79	3–33	28.5	26.79	8.67	10.9–42	<0.001*
WMLV (cm^3^)		12.13	22.33	20.66	4.37–94.21	2.87	4.85	4.68	0.30–17.84	< 0.001*
Gender (% male)		34.9				28.6				0.534
Ethnicity (%)										0.355
	Malay	23.3				9.5				
	Chinese	58.1				73.8				
	Indian	14.0				11.9				
	Others	4.7				4.8				
Diabetes (%)		32.6				19.5				0.174
Hypertension (%)		62.8				26.8				0.001*
Stroke (%)		9.3				9.8				0.335
OH (%)		41.9				15.0				0.007*
Syncopal Events (%)		34.9				12.5				0.017*
CSS (%)		9.3				2.5				0.196
VI (%)		20.9				10.0				0.17
OA (%)		11.6				12.5				0.904
PN (%)		9.3				2.5				0.196

*p<0.05

CSS, carotid sinus syndrome; FR, functional reach; OA, osteoarthritis; OH, orthostatic hypotension; PN, peripheral neuropathy; TUG, timed-up-and-go; VI, visual impairment; WMLV, white matter lesion volume

## Results

The demographic characteristics of fallers and non-fallers are presented in Table 1. Fallers ranged between 65 and 83 years in age (mean, 73.21; standard deviation (SD), 4.26), whereas the non-fallers ranged between 65 and 84 years in age (mean, 71.52; SD 4.55). Both fallers and non-fallers were predominantly female (65.1% and 71.4% respectively). There was no significant difference in the gender and ethnicity of the fallers versus non-fallers. The mean age and timed-up-and-go (TUG) were higher in the fallers group compared to non-fallers, whereas FR was lower in fallers compared to non-fallers (mean, 19.97 cm; SD 7.79. vs mean, 26.79 cm; SD 8.67). Fallers had significantly higher white matter lesion volume than non-faller (mean, 22.33 cm ^3^; SD 20.66 vs mean, 4.85 cm ^3^; SD 4.68).

Tested individually, clinical variables that demonstrated statistical significances were hypertension, orthostatic hypotension and syncopal events. All the clinical variables with significant differences showed higher fractional values in the fallers group compared to the non-fallers. Table 2 describes the history of falls of the fallers and non-fallers group. Most of the fallers had one indoor or outdoor fall with the presence of several associated symptoms such as vertigo, presyncope, dizziness, loss of consciousness, slips or trips or visual problems.

**Table 2:** History of falls for fallers and non-fallers group.

Characteristics		Percentages	
		Fallers (n = 44)	Non-fallers (n = 41)
Indoor Falls			
	No fall	43.2	100
	One fall	29.5	0
	More than one fall	27.3	0
Outdoor Falls			
	No fall	36.4	100
	One fall	34.1	0
	More than one fall	29.5	0
Injury		59.1	0
Medical attention		27.3	0
Hospital admission		13.6	0
Vertigo		11.4	14.6
Presyncope		11.4	14.6
Dizziness		27.3	29.3
LOC		13.6	0
Slips or trips		50	22
Visual Problems		27.3	29.3

LOC, loss of consciousness

**Fig 2:**
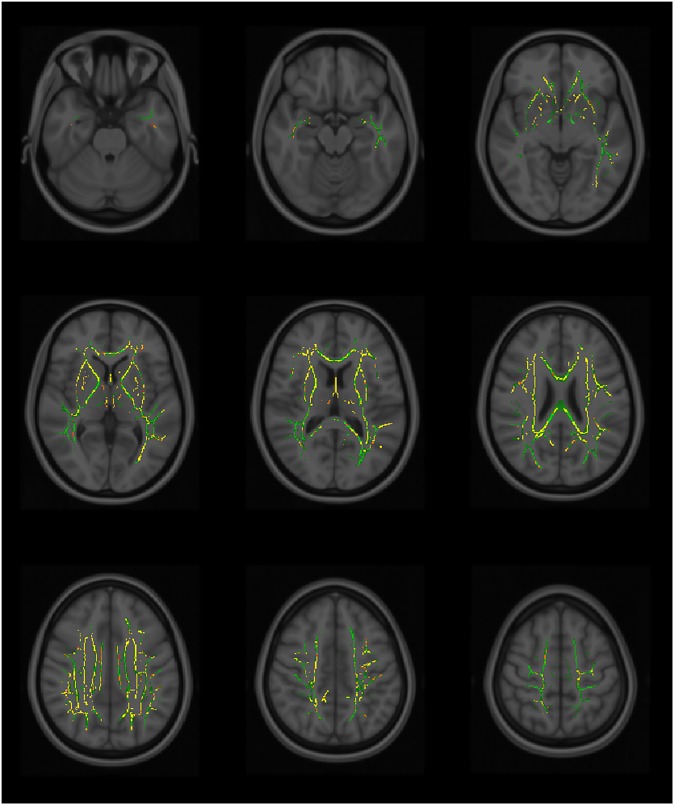
The tract-based spatial statistics (TBSS). The tract-based spatial statistics (TBSS) showing mean diffusivity (MD) voxels of the white matter tracts that demonstrated significant differences between the fallers and non-fallers. TBSS skeleton (green); p <0.05 (red-yellow); significance levels gradient (red < orange < yellow).

**Fig 3:**
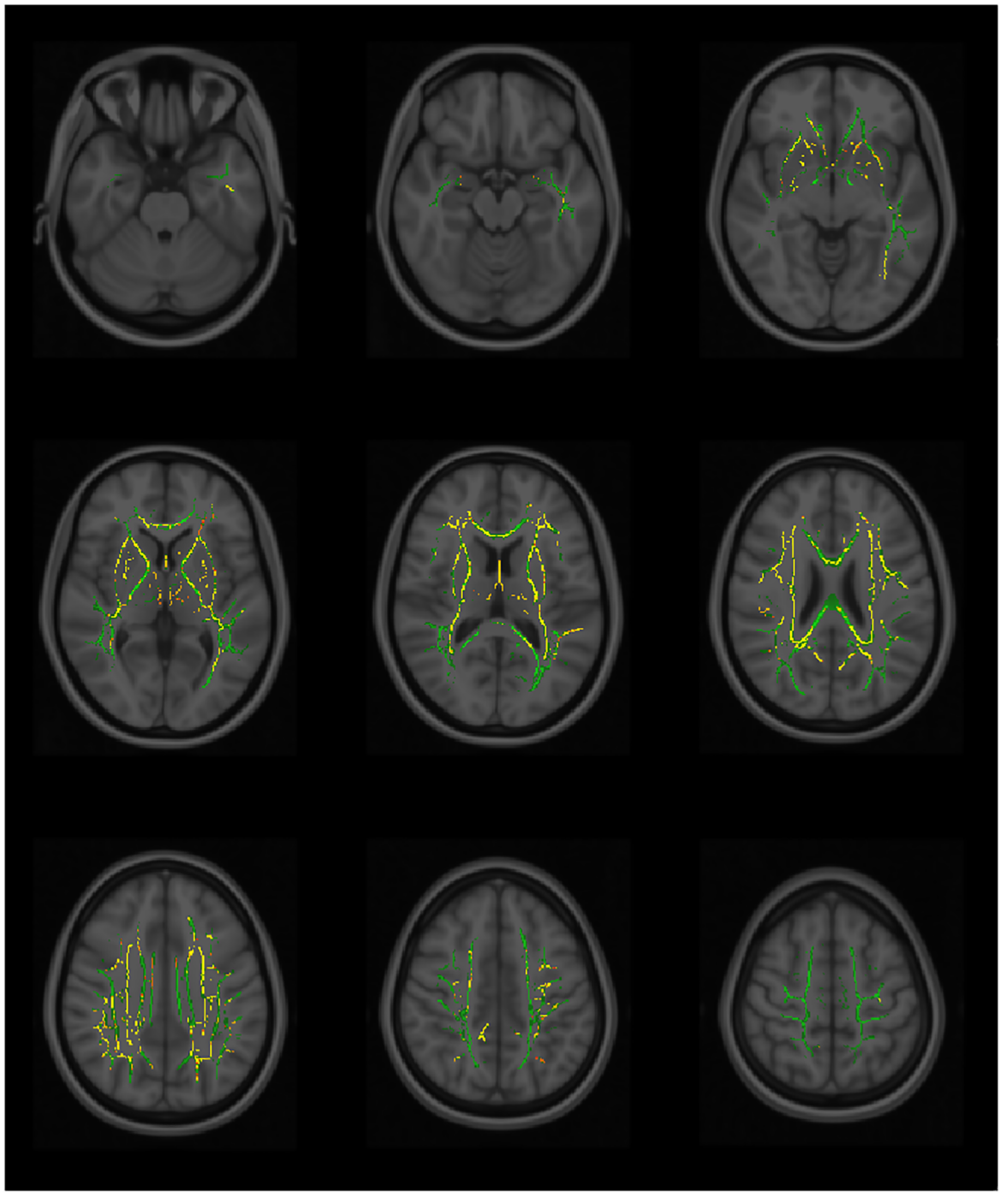
The tract-based spatial statistics (TBSS). The tract-based spatial statistics (TBSS) showing axial diffusivity (AD) voxels of the white matter tracts that demonstrated significant differences between the fallers and non-fallers. TBSS skeleton (green); p <0.05 (red-yellow); significance levels gradient (red < orange < yellow).

**Fig 4:**
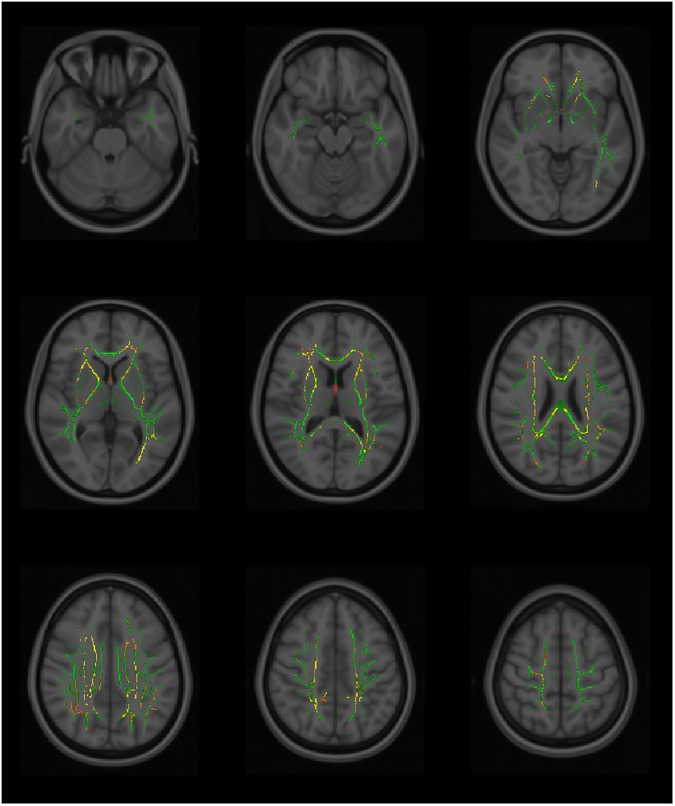
The tract-based spatial statistics (TBSS). The tract-based spatial statistics (TBSS) showing radial diffusivity (RD) voxels of the white matter tracts that demonstrated significant differences between the fallers and non-fallers. TBSS skeleton (green); p <0.05 (red-yellow); significance levels gradient (red < orange < yellow).

Using TBSS, significant differences in the white matter tracts are seen between the fallers versus non-fallers in the MD and AD values genu/body/splenium of corpus callosum, fornix, anterior /posterior/ retrolenticular part of the internal capsules, external capsule, anterior/posterior/superior corona radiata, posterior thalamic radiation, superior longitudinal fasciculus, superior fronto-occipital fasciculus and tapetum (Figs 2 & 3 and S1 Table). The RD values showed significant differences in the genu/body/splenium of corpus callosum, anterior part of the internal capsule, anterior/posterior/superior corona radiata, external capsule, superior fronto-occipital fasciculus and both tapetum (Fig 4). The white matter fibers involved are mainly the projection and commissural bundles. The FA values of the tracts however did not demonstrate significant differences between fallers and non-fallers.

**Fig 5:**
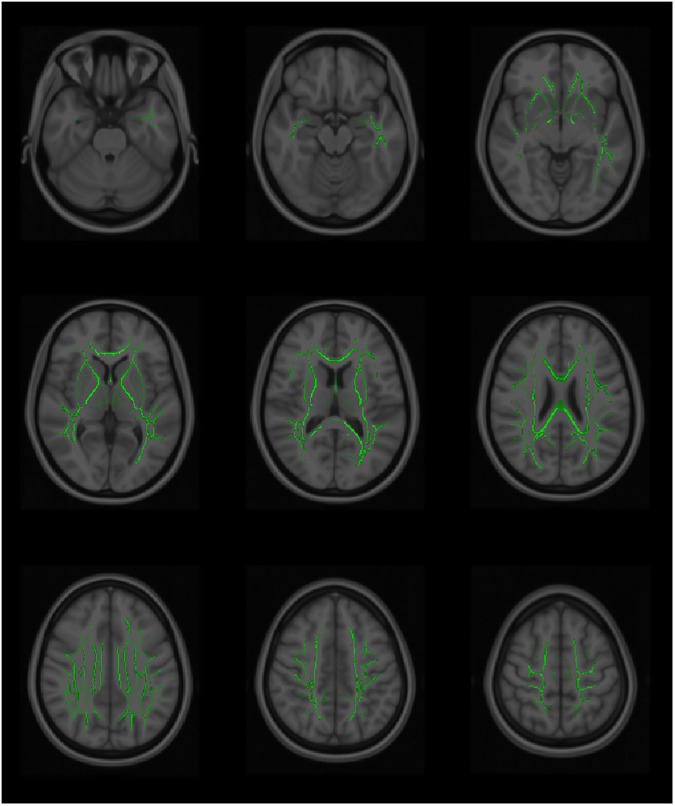
The tract-based spatial statistics (TBSS). The tract-based spatial statistics (TBSS) showing medial diffusivity (MD) voxels of the white matter tracts between the fallers and non-fallers, taking into account age, functional reach (FR), white matter lesion volume, hypertension and orthostatic hypotension as covariates. TBSS skeleton (green).

To take into account all the confounding factors that may contribute towards falls, we then performed a multiple logistic regression analysis. Clinical variables known to be related to falls which demonstrated significant differences in univariate analysis (age, TUG, FR, WMLV, hypertension, orthostatic hypotension, and syncopal events) were entered for stepwise backward elimination of the non-significant variables. The final logistic regression model obtained showed that only functional reach (FR), white matter lesion volume, hypertension and orthostatic hypotension demonstrated statistical significant differences between fallers and non-fallers, with age retained in the model although no significance is shown (Table 3). Age and the four clinical variables were then incorporated as covariates in the repeated TBSS analysis. No significant difference was found after rerunning the TBSS analysis taking into account the covariates (Fig 5).

**Table 3:** The logistic regression analysis of the covariates using backward LR selection.

				95% CI for Odds Ratio		
Step		B (SE)	Odds Ratio	Lower	Upper	p-value
First step	Age	-0.209 (0.108)	0.812	0.657	1.003	0.054
	TUG	0.036 (0.069)	1.037	0.907	1.186	0.596
	FR	-0.128 (0.054)	0.880	0.791	0.979	0.019*
	WML	0.286 (0.081)	1.332	1.135	1.562	<0.001*
	HPT	1.981 (0.860)	7.250	1.343	39.154	0.021*
	OH	1.489 (0.888)	4.431	0.778	25.240	0.094
	VS	1.424 (1.098)	4.156	0.483	35.752	0.195
	Constant	13.347 (7.611)				
Final step	Included					
	Age	-0.183 (0.103)	0.833	0.681	1.020	0.076
	FR	-0.151 (0.050)	0.860	0.779	0.949	0.003*
	WML	0.276 (0.076)	1.318	1.136	1.530	<0.001*
	HPT	1.750 (0.821)	5.757	1.152	28.761	0.033*
	OH	1.903 (0.850)	6.707	1.268	35.486	0.025*
	Constant	12.845 (7.319)				

FR, functional reach; HPT, hypertension; OH, orthostatic hypotension; TUG, Timed Up-and-Go; VS, vasovagal syncope; WML, white matter lesion

*p<0.05

## Discussion

This is the first study utilising DTI for whole brain white matter tract analysis while taking into account confounding factors of fallers in an ageing multi-racial Asian population. DTI has been shown to be a reliable technique to detect microstructural abnormalities in the individual white matter tracts [22]. Using whole-brain TBSS analysis, we found significant differences in MD and AD values for various white matter tracts between fallers and non-fallers. Significant tracts included the projection fibers and commissural bundles.

Previous studies evaluated the relationship between motor disturbances, grades and DTI metrics in Parkinson’s patients and found association between motor disturbances and the DTI abnormalities in the body of the corpus callosum [23, 24]. Several studies have concluded that older people with increased risk of falls have gait and DTI abnormalities in a number of WM tracts, which include genu and splenium of the corpus callosum, corticospinal tract, anterior limb of internal capsule medial frontal and parietal subcortical pathways, and frontal-parietal-temporal longitudinal pathways [11, 12, 25, 26]. Further studies [27–29] have reported that increases in AD are linked to axonal damage which unveils potential structural changes associated with falls in older adults. An AD change not accompanied by FA changes is thought to represent widespread tissue damage, gross tissue loss, increased extracellular space as a result of axonal atrophy secondary to Wallerian degeneration [30, 31]. Increased MD in DTI measurements are considered non-specific and are associated with inflammation or oedema [32].

The microstructural changes in major WM tracts are associated with age-related changes in adults older than 50 years [33]. WM degeneration appears to be detectable in even healthy older individuals while aging has been shown to be accompanied by functional decline even in the absence of disease [34]. The decline in WM microstructural organization was reported to be associated with lower gait stability [35–37]. In our study, age did not contribute significantly to the outcome of falls when taking into account the other contributing factors.

Aside from the clinical parameters such as FR, hypertension and orthostatic hypotension, the imaging parameter of significant values is total WML volume in fallers. WML affect the information processing speed and executive function [38]. WML had also shown to increase the risk of falls in the elderly population. Many cross sectional and longitudinal studies have shown that white matter changes within the brain are associated with falls, gait and balance disturbances [9–11].

To understand the confounding effects of the clinical and imaging variables towards the outcomes (fallers and non-fallers), a multiple binary logistic regression analysis was performed, which identified functional reach, WML volume, hypertension, and orthostatic hypotension as independent predictors of falls. Repeated TBSS analysis accounting for age and these four variables demonstrated that DTI metrics did not contribute significantly to the outcomes independent of the covariates, which identifies that the difference in DTI metrics between fallers and non-fallers were mediated by the differences in dynamic balance, WML volume and blood pressure drop with posture change between the two groups [39].

Hemodynamic changes related to orthostatic hypotension were reported to be associated with white matter changes and white matter hyperintensities volume in patients with neurodegenerative disorder [40, 41]. Another factor that was identified to have an independent effect on falls is the presence of hypertension as comorbidity. Hypertension is a known vascular risk factor associated with WML or leukoaraiosis. There is increasing evidence that the underlying pathophysiology of leukoaraiosis is due to microvascular disease secondary to hypertension resulting in chronic cerebral ischemia [42, 43].

Several limitations were apparent in our study. Due to the presence of susceptibility artefacts at the brainstem and cerebellum in our DTI datasets, the white matter tracts assessment for a number of subjects had been eliminated from our analyses. Our study was limited by its relatively small sample size and cross-sectional design, therefore limiting any causal inference. The assessment of falls was retrospective based on patient history that may be inaccurate. A larger, prospective study should now be conducted to evaluate WM integrity and their correlation with new falls or new motor disturbances in older adults.

## Conclusion

Overall DTI data suggests that there are changes in the white matter micro-structural integrity in projection fibers and commissural bundles in fallers. However, DTI is not a singular factor that contributes independently to the fall outcomes in the presence of functional reach, WML volume, hypertension, and orthostatic hypotension. This novel finding importantly highlights the potentially synergistic relationship between postural blood pressure fluctuations, balance disorders and structural abnormalities observed among fallers. Prospective evaluation of this relationship is required to establish the potential causal relationship between these factors.

## Supporting information

S1 Table The TBSS analysis. The tract-based spatial statistics (TBSS) of the DTI metrics.
(XLSX)

## Abbreviations

ACC: anterior cingulate cortex
AD: axial diffusivity
DLPFC: dorsolateral prefrontal cortex
DTI: diffusion tensor imaging
FA: fractional anisotropy
FLAIR: fluid attenuation inversion recovery
FSPGR: fast spoiled gradient echo
GRE: gradient recalled echo
ICBM: International Consortium of Brain Mapping
IQR: interquartil range
MD: mean diffusivity
MRI: magnetic resonance imaging
MyFAIT: Malaysian Falls Assessment and Intervention Trial
RD: radial diffusivity
RF: radiofrequency
SD: standard deviation
SE: spin echo
TBBS: tract-based spatial statistics
WM: white matter
WML: white matter lesions
WMT: white matter tracts

## References

[1] Lord SR (2007) Falls in older people Risk factors and stategies for prevention. Cambridge University Press, Cambridge United Kingdom

[2] Bloem BR, Steijns JAG, Smits-Engelsman BC (2003) An update on falls. Curr Opin Neurol 16:15–26 doi: 10.1097/01.wco.0000053580.70044.70 12544853 10.1097/01.wco.0000053580.70044.70

[3] Tan MP, Kamaruzzaman SR, Zakaria MI, Chin AV, Poi PJH (2016) Ten-year mortality in older patients attending the emergency department after a fall. Geriatrics & Gerontology International 16(1): p. 111–117. 25613422 10.1111/ggi.12446

[4] Englander F, Hodson TJ, Terregrossa RA (1996) Economic dimensions of slip and fall injuries. J Forensic Sci 41:733–746 8789837

[5] Axer H, Axer M, Sauer H, Witte OW, Hagemann G (2010) Falls and gait disorders in geriatric neurology. Clin Neurol Neurosurg 112:265–74 doi: 10.1016/j.clineuro.2009.12.015 20089351 10.1016/j.clineuro.2009.12.015

[6] Ambrose AF, Paul G, Hausdorff JM (2013) Risk factors for falls among older adults: a review of the literature. Maturitas 75:51–61 doi: 10.1016/j.maturitas.2013.02.009 23523272 10.1016/j.maturitas.2013.02.009

[7] Baezner H, Blahak C, Poggesi A, et al (2008) Association of gait and balance disorders with age-related white matter changes: the LADIS study. Neurology 70:935–42 doi: 10.1212/01.wnl.0000305959.46197.e6 18347315 10.1212/01.wnl.0000305959.46197.e6

[8] Blahak C, Baezner H, Pantoni L, et al (2009) Deep frontal and periventricular age related white matter changes but not basal ganglia and infratentorial hyperintensities are associated with falls: cross sectional results from the LADIS study. J Neurol Neurosurg Psychiatry 80:608–613 doi: 10.1136/jnnp.2008.154633 19204027 10.1136/jnnp.2008.154633

[9] De Laat KF, Van Norden AG, Gons RA, et al (2011) Diffusion tensor imaging and gait in elderly persons with cerebral small vessel disease. Stroke 42:373–379 doi: 10.1161/STROKEAHA.110.596502 21193751 10.1161/STROKEAHA.110.596502

[10] Wakana S, Jiang H, Zijl PCM Van (2003) Fiber Tract–based Atlas of Human White Matter Anatomy. Radiology 230:77–87 doi: 10.1148/radiol.2301021640 14645885 10.1148/radiol.2301021640

[11] Bhadelia RA, Price LL, Tedesco KL, et al (2009) Diffusion tensor imaging, white matter lesions, the corpus callosum, and gait in the elderly. Stroke 40:3816–20 doi: 10.1161/STROKEAHA.109.564765 19797696 10.1161/STROKEAHA.109.564765 PMC3401013

[12] Koo BB, Bergethon P, Qiu WQ, et al (2012) Clinical prediction of fall risk and white matter abnormalities: a diffusion tensor imaging study. Arch Neurol 69:733–738 doi: 10.1001/archneurol.2011.2272 22332181 10.1001/archneurol.2011.2272 PMC4443844

[13] Tan PJ, Khoo EM, Chinna K, Hill KD, Poi PJH, Tan MP (2014) An individually-tailored multifactorial intervention program for older fallers in a middle-income developing country: Malaysian Falls Assessment and Intervention Trial (MyFAIT). BMC Geriatr 14:78 doi: 10.1186/1471-2318-14-78 24951180 10.1186/1471-2318-14-78 PMC4080753

[14] Zia A, Kamaruzzaman SB, Tan MP (2015) Blood pressure lowering therapy in older people: Does it really cause postural hypotension or falls? Postgraduate Medicine 127(2): p. 186–193. doi: 10.1080/00325481.2015.996505 25622817 10.1080/00325481.2015.996505

[15] Smith SM, Jenkinson M, Johansen-Berg H, et al (2006) Tract-based spatial statistics: voxelwise analysis of multi-subject diffusion data. Neuroimage 31:1487–505 doi: 10.1016/j.neuroimage.2006.02.024 16624579 10.1016/j.neuroimage.2006.02.024

[16] TBSS/ UserGuide. In: FMRIB Softw. Libr. v5.0. (FSL). Available via http://fsl.fmrib.ox.ac.uk/fsl/fslwiki/TBSS/UserGuide. Accessed 24 Feb 2015

[17] Gaser’s, C., VBM8 toolbox. In: Struct. Brain Mapp. Gr. Available via http://dbm.neuro.uni-jena.de/vbm/. Accessed 24 Feb 2015.

[18] Statistical Parametric Mapping software package version 8 (SPM8). Available via http://www.fil.ion.ucl.ac.uk/spm/software/spm8/, 2009. Accessed 24 Feb 2015.

[19] Lesion Segmentation Toolbox (LST). In: Struct. Brain Mapp. Gr. Available via http://dbm.neuro.uni-jena.de/software/lst/. Accessed 24 Feb 2015.

[20] Schmidt P, Gaser C, Arsic M, Buck D, Forschler, et al (2012) An automated tool for detection of FLAIR-hyperintense white-matter lesions in Multiple Sclerosis. NeuroImage 59(4): p. 3774–3783. doi: 10.1016/j.neuroimage.2011.11.032 22119648 10.1016/j.neuroimage.2011.11.032

[21] Maldjian JA, Whitlow CT, Saha BN, Kota G, Vandergriff C, Davenport EM, et al (2013) Automated White Matter Total Lesion Volume Segmentation in Diabetes. American Journal of Neuroradiology 34(12): p. 2265–2270. doi: 10.3174/ajnr.A3590 23868156 10.3174/ajnr.A3590 PMC4038900

[22] Nucifora PGP, Verma R, Lee SK, Melhem ER (2007) Diffusion-Tensor MR Imaging and Tractography: Exploring Brain Microstructure and Connectivity. Radiology 245(2): p. 367–384. doi: 10.1148/radiol.2452060445 17940300 10.1148/radiol.2452060445

[23] Lee SJ, Kim JS, Lee KS, et al (2009) The severity of leukoaraiosis correlates with the clinical phenotype of Parkinson’s disease. Arch Gerontol Geriatr 49:255–259 doi: 10.1016/j.archger.2008.09.005 18977043 10.1016/j.archger.2008.09.005

[24] Chan LL, Ng KM, Rumpel H, Fook-Chong S, Li HH, Tan EK (2014) Transcallosal diffusion tensor abnormalities in predominant gait disorder parkinsonism. Park Relat Disord 20:53–59 10.1016/j.parkreldis.2013.09.017 24126023

[25] De Laat KF, Tuladhar AM, Van Norden AGW, Norris DG, Zwiers MP, De Leeuw FE (2011) Loss of white matter integrity is associated with gait disorders in cerebral small vessel disease. Brain 134(1): p. 73–83. 21156660 10.1093/brain/awq343

[26] Kafri M, Sasson E, Assaf Y, Balash Y, Aiznstein O, Hausdorff JM, et al (2012) High-level gait disorder: associations with specific white matter changes observed on advanced diffusion imaging. J Neuroimaging 2012(23): p. 39–46. 10.1111/j.1552-6569.2012.00734.x PMC3514556 22928624

[27] Roosendaal SD, Geurts JJG, Vrenken H, et al (2009) Regional DTI differences in multiple sclerosis patients. Neuroimage 44:1397–1403 doi: 10.1016/j.neuroimage.2008.10.026 19027076 10.1016/j.neuroimage.2008.10.026

[28] Metwalli NS, Benatar M, Nair G, Usher S, Hu X, Carew JD (2010) Utility of axial and radial diffusivity from diffusion tensor MRI as markers of neurodegeneration in amyotrophic lateral sclerosis. Brain Res 1348:156–164 doi: 10.1016/j.brainres.2010.05.067 20513367 10.1016/j.brainres.2010.05.067

[29] Hasan KM, Halphen C, Boska MD, Narayana PA (2008) Diffusion tensor metrics, T2 relaxation, and volumetry of the naturally aging human caudate nuclei in healthy young and middle-aged adults: Possible implications for the neurobiology of human brain aging and disease. Magn Reson Med 59:7–13 doi: 10.1002/mrm.21434 18050345 10.1002/mrm.21434

[30] Veeramuthu V, Narayanan NV, Tan LK, et al (2015) Diffusion tensor imaging parameters in mild traumatic brain injury and its correlation with early neuropsychological impairment: A longitudinal study. J Neurotrauma 13:1–13 10.1089/neu.2014.3750 PMC4589266 25952562

[31] Alves GS, Oertel Knöchel V, Knöchel C, et al (2015) Integrating retrogenesis theory to alzheimer’s disease pathology: Insight from DTI-TBSS investigation of the white matter microstructural integrity. Biomed Res Int 2015:1–11 10.1155/2015/291658 PMC4320890 25685779

[32] Alexander AL, Lee JE, Lazar M, Field AS (2007) Diffusion tensor imaging of the brain. Neurotherapeutics 4:316–329 doi: 10.1016/j.nurt.2007.05.011 17599699 10.1016/j.nurt.2007.05.011 PMC2041910

[33] Sexton CE, Walhovd KB, Storsve AB, Tamnes CK, Westkye LT, Johansen-Berg H, et al (2014) Accelerated Changes in White Matter Microstructure during Aging: A Longitudinal Diffusion Tensor Imaging Study. The Journal of Neuroscience 34(46): p. 15425–15436. doi: 10.1523/JNEUROSCI.0203-14.2014 25392509 10.1523/JNEUROSCI.0203-14.2014 PMC4228140

[34] Griebe M, Forster A, Wessa M, Rossmanith C, Bazner H, Sauer T, et al (2011) Loss of callosal fibre integrity in healthy elderly with age-related white matter changes. Journal of Neurology 258(8): p. 1451–1459. doi: 10.1007/s00415-011-5956-6 21340519 10.1007/s00415-011-5956-6

[35] Bruijn SM, Van Impe A, Duysens J, Swinnen SP (2014) White matter microstructural organization and gait stability in older adults. Frontiers in Aging Neuroscience 6(104). 10.3389/fnagi.2014.00104 PMC4051125 24959139

[36] Vercruysse S, Leunissen I, Vervoot G, Vandenberghe W, Swinnen S, Nieuwboer A (2015) Microstructural changes in white matter associated with freezing of gait in Parkinson's disease. Movement Disorders 30(4): p. 567–576. doi: 10.1002/mds.26130 25640958 10.1002/mds.26130

[37] Iseki K, Fukuyama H, Oishi N, Tomimoto H, Otsuka Y, Nankaku M, et al (2015) Freezing of gait and white matter changes: a tract-based spatial statistics study. Journal of Clinical Movement Disorders 2: p. 1 doi: 10.1186/s40734-014-0011-2 26788337 10.1186/s40734-014-0011-2 PMC4711070

[38] Prins ND, van Dijk EJ, Heijer T, Vermeer SE, Jolles J, Koudstaal PJ, et al (2005) Cerebral small-vessel disease and decline in information processing speed, executive function and memory. Brain 128(9): p. 2034–2041. 15947059 10.1093/brain/awh553

[39] Ballard C, O Brien J, Barber BOB, Scheltens O, Shaw F, McKeith IAN, et al (2000) Neurocardiovascular Instability, Hypotensive Episodes, and MRI Lesions in Neurodegenerative Dementia. Annals of the New York Academy of Sciences 903(1): p. 442–445. 10818535 10.1111/j.1749-6632.2000.tb06396.x

[40] Oh YS, Kim JS, and Lee KS (2013) Orthostatic and Supine Blood Pressures Are Associated with White Matter Hyperintensities in Parkinson Disease. Journal of Movement Disorders 6(2): p. 23–27. doi: 10.14802/jmd.13006 24868422 10.14802/jmd.13006 PMC4027644

[41] Colloby SJ, Vasudev A, O Brien JT, Firbank MJ, Parry SW, Thomas AJ (2011) Relationship of orthostatic blood pressure to white matter hyperintensities and subcortical volumes in late-life depression. British Journal of Psychiatry 199(5): p. 404–410. doi: 10.1192/bjp.bp.110.090423 21903666 10.1192/bjp.bp.110.090423

[42] Basile A.M, Pantoni L, Pracucci G, Asplund K, Chabriat H, Erkinjuntti T, et al (2006) Age, Hypertension, and Lacunar Stroke Are the Major Determinants of the Severity of Age-Related White Matter Changes. Cerebrovascular Diseases 21(5–6): p. 315–322. doi: 10.1159/000091536 16490940 10.1159/000091536

[43] Pantoni L (2002) Pathophysiology of Age-Related Cerebral White Matter Changes. Cerebrovascular Diseases 13(suppl 2)(Suppl. 2): p. 7–10. 10.1159/000049143 11901236

